# The influence of gaze direction on food preferences

**DOI:** 10.1038/s41598-019-41815-9

**Published:** 2019-04-03

**Authors:** Apoorva Rajiv Madipakkam, Gabriele Bellucci, Marcus Rothkirch, Soyoung Q. Park

**Affiliations:** 1 Institute for Psychology, University of Lübeck, Ratzeburger Allee 160, 23562 Lübeck, Germany; 2 Charité-Universitätsmedizin Berlin, Corporate member of Freie Universität Berlin, Humboldt-Universität zu Berlin, and Berlin Institute of Health, Neuroscience Research Center, 10117 Berlin, Germany; 3 Decision Neuroscience and Nutrition, German Institute of Human Nutrition (DIfE), Potsdam-Rehbruecke, Germany

## Abstract

In our information-rich environment, the gaze direction of another indicates their current focus of attention. Following the gaze of another, results in gaze-evoked shifts in joint attention, a phenomenon critical for the functioning of social cognition. Previous research in joint attention has shown that objects that are attended by another are more liked than ignored objects. Here, we investigated this effect of gaze-cueing on participants’ preferences for unknown food items. Participants provided their willingness to pay (WTP), taste and health preferences for food items before and after a standard gaze-cueing paradigm. We observed a significant effect of gaze-cueing on participants’ WTP bids. Specifically, participants were willing to pay more money for the food items that were looked at by another person. In contrast, there was a decrease in preference for the food items that were ignored by another person. Interestingly, this increase in WTP occurred without participants’ awareness of the contingency between the cue and target. These results highlight the influence of social information on human choice behavior and lay the foundation for experiments in neuromarketing and consumer decision making.

## Introduction

Every day we make multiple food choices. Such choices, which reflect our eating patterns, are strongly influenced by not only our physical, but also social environment^1,2^. Poor food choices, such as strong preferences for high caloric food, is one of the main contributors to the growing problem of obesity^3–5^. Attention to food is a primary determinant of preference formation, food consumption and even obesity^6–8^. In line with this, overt attention has been shown to correlate strongly with choice behaviour, i.e., participants spend more time fixating an item before choosing it^9^. A potent signal of overt attention is eye gaze direction. Eye gaze plays an important role in human communication, indicating a person’s emotions, thoughts and intentions^10^. In particular, people tend to follow the gaze of another person, as it provides them with information about stimuli in their environment. Such gaze-evoked shifts in attention are mostly reflexive, i.e., outside voluntary control^11^ and occur even when the gaze cue is subliminal^12–14^, resulting in a faster processing of targets in the gazed-at location than the ignored location^15,16^. Importantly, previous research has shown that another’s gaze not only shifts the observer’s attention, but also impacts their affective evaluations of the gazed-at objects^12,17,18^. For instance, objects that are the focus of another’s attention are not only preferred over unattended ones^19^, they are also better remembered than the ignored objects^20^. Importantly, this increase in liking of an object from the gaze-evoked shift in attention was specifically observed for eye gaze cues and not for other cues causing similar shifts in attention, whether they were social, like pointing hands^21^, or non-social, like arrows^19^. This emphasizes the special role of eye gaze information in communication and decision-making.

While there is a wealth of research supporting such an increase in likability of objects attended by others, it is unknown whether evaluations of food items are also affected by another’s gaze. Such a question is highly relevant to a variety of fields including consumer psychology, food science and food policy. Indeed, recent eye-tracking evidence suggests that the presence of gaze cues in a print advertisement impacts the time spent looking at the product of interest. In other words, more time was spent looking at the product when the person in the advertisement was also gazing at the product^22,23^. Furthermore, some evidence points to the influence of social-affective context and social cues in shaping food preferences in children^24,25^. For instance, children’s preferences for snack items was increased when the snack item was presented as a reward or with adult attention. However, no such increase in preference was found when the snack was presented in a non-social context^24^. Similarly, Harpers and Sander showed that children readily tried an unfamiliar food when an adult was also shown eating it, than when it was presented alone^26^. These studies thus show the influence of social-affective context in shaping food preferences.

In the current study, we investigated whether food preferences and their evaluations change as a function of gaze-evoked shifts in attention. As dependent variables for food evaluation, we assessed the tastiness and healthiness of food items, that have been shown to drive others’ choices^27^. In fact, the tastiness of a food item has been shown to automatically capture visual attention^28^. Moreover, we investigated the preference for a product measuring the individual, economic value ascribed to it. To this end, we employed the willingness-to-pay (WTP) paradigm, in which participants indicated the maximum amount they were willing to pay for the presented food items. Preferred food items are associated with a higher WTP^29,30^. Based on the accumulating evidence for the increase in likability for objects that are the focus of another’s attention in comparison to those that are unattended and ignored, we hypothesized that participants’ preferences for food items that are looked at by another person increases in comparison to those that are not. Furthermore, we used eye-tracking to determine whether participants increase in preference to the food item was influenced by the gaze-evoked shifts in attention. Specifically, the dwell time or the time spent looking at the product was used as an index of overt attention.

## Results

### Analysis of behavioural data

#### Reaction Times

**Figure 1:**
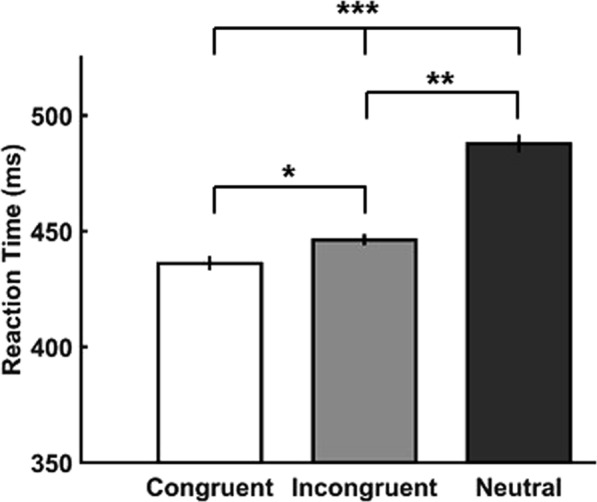
Gaze cueing effect. Significant main effect of congruence on reaction times to detect the snack product. Asterisks (*) indicate statistically significant differences: ^*^p < 0.05; ^**^p < 0.01; ^***^p < 0.001, Bonferroni corrected. Error bars denote within-subject SEM.

As a first step, we analysed participants’ performance and time to detect the food stimulus in the gaze-cueing task. All participants performed the task during the cueing phase with high accuracy (*M* = 88.7% ± 3.5 SEM). We did not observe any differences in participants’ task performance between the three cueing conditions (*F*(2,62) < 1, *p* > 0.5). In line with the literature on gaze cueing^15,16,31,32^, we found a significant main effect of congruence on reaction times (*F*(1.56,48.3) = 48.74, *p* < 0.001; degrees of freedom corrected with Greenhouse-Geisser for violation of sphericity, ε = 0.71). Specifically, reaction times in the congruent condition were faster (*M* = 436.1 ms ± 3.1 SEM) than those in the incongruent (*M* = 446.2 ms ± 2.4 SEM) and neutral conditions (*M* = 487.8 ms ± 3.9 SEM). Bonferroni-corrected post-hoc t-tests revealed significant differences in reaction times between the congruent and incongruent condition (*t*(31) = −2.54, *p*_*corr*_ = 0.048; *p*_*corr*_ refers to the Bonferroni corrected *p* value), congruent and neutral condition (*t*(31) = −7.79, *p*_*corr*_ = 0.003) and incongruent and neutral condition (*t*(31) = −7.30, *p*_*corr*_ = 0.003; Fig. 1).

#### Ratings

**Figure 2:**
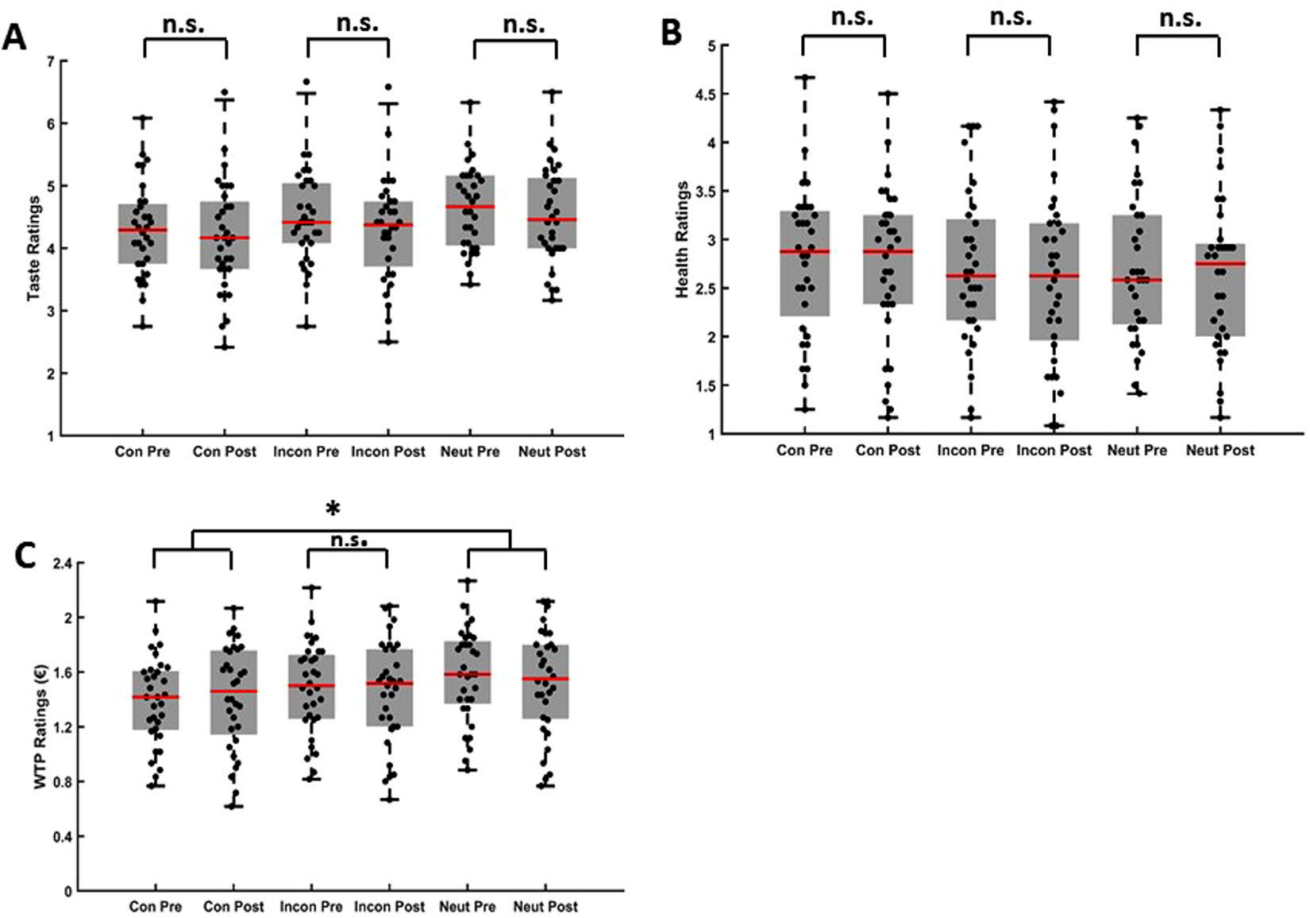
Participants’ pre and post ratings. (**A**) Taste, (**B**) health ratings and (**C**) willingness-to-pay (WTP) bids. A significant difference in the change in ratings in the congruent condition to the change in ratings in the neutral condition was observed. Asterisks (*) indicate statistically significant differences: n.s., not significant; ^*^p < 0.05; red bars indicate the median ratings.

To investigate whether the direction of gaze of the cue influenced participants’ food preferences, a 2 × 3 repeated measures Analysis of Variance (ANOVA) with time (pre/post) and gaze (congruent/incongruent/neutral) as within-subject factors was performed on participants’ raw ratings for taste (Fig. 2A), health (Fig. 2B) and WTP (Fig. 2C). For participants’ taste ratings there was a significant main effect of time (*F*(1,31) = 9.13, *p* = 0.005, *η²* = 0.23) and a significant main effect of gaze (*F*(2,62) = 17.65, *p* < 0.001, *η²* = 0.36). There was no interaction between gaze x time (*F*(2,62) = 0.84, *p* = 0.44, *η²* = 0.03). Post-hoc t-tests to explore the significant main effect of time revealed that participants pre-ratings (*M* = 4.47 ± 0.12 SEM) were higher than their post-ratings (*M* = 4.34 ± 0.12 SEM; *t*(31) = 3.02, *p* = 0.005). Furthermore, participants’ ratings in the neutral condition (*M* = 4.59 ± 1.27) were higher than in the congruent (*M* = 4.23 ± 0. 14 SEM; *t*(31) = 6.5, *p*_*corr*_ = 0.003) and incongruent conditions (*M* = 4.40 ± 0.14 SEM; *t*(31) = 3.31, *p*_*corr*_ = 0.006). Participants’ taste ratings in the congruent condition were lower than in the incongruent condition (*t*(31) = −2.5, *p*_*corr*_ = 0.06).

**Figure 3:**
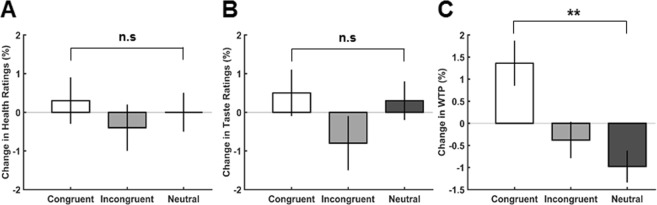
Change in Ratings. Mean change in (**A**) health, (**B**) taste ratings and (**C**) willingness-to-pay (WTP) bids. A significant effect of congruence was observed in participants’ WTP ratings with an increase for the congruent snack products. Asterisks (*) indicate statistically significant differences: n.s., not significant; ^*^p < 0.05; ^**^p < 0.01. Error bars denote within-subject SEM.

The 2 × 3 repeated measures ANOVA for participants’ health ratings revealed no significant main effect of time (*F*(1,31) = 2.92, *p* = 0.10), main effect of gaze (*F*(2,62) = 0.65, *p* = 0.52) or interaction between gaze x time (*F*(2,62) < 1, *p* > 0.5). Interestingly however, the 2 × 3 ANOVA with the factors time and gaze for participants’ WTP bids revealed a significant main effect of gaze (*F*(2,62) = 70.40, *p* < 0.001, *η²* = 0.69) and importantly, a significant interaction effect of gaze-by-time (*F*(2,62) = 5.31, *p* = 0.007, *η²* = 0.15) suggesting that the gaze cue had an effect on participants’ WTP bids. There was no main effect of time (*F*(1,31) = 0.59, *p* = 0.45). Post-hoc t-tests to explore the significant main effect of gaze revealed that participants bids were lowest in the congruent condition (*M* = 1.20€ ± 0.06 SEM) compared to the incongruent (*M* = 1.26€ ± 0.06 SEM; t(31) = −4.8, *p*_*corr*_ < 0.003) and neutral conditions (*M* = 1.35 € ± 0.06 SEM; *t*(31) = −11.2, *p*_*corr*_ < 0.003). Participants ratings in the neutral condition were higher than in the incongruent condition (*t*(31) = 7.5, *p*_*corr*_ < 0.003). Bonferroni corrected post hoc t-tests to explore the significant interaction effect were however not significant (congruent pre-rating (*M* = 1.19 € ± 0.05 SEM) vs. congruent post rating (*M* = 1.22 € ± 0.07; *t*(31) = −1.03, p = 0.31); incongruent pre-rating (*M* = 1.28 € ± 0.06 SEM) vs. incongruent post rating (*M* = 1.25€ ± 0.07; *t*(31) = 1.10, p = 0.28); neutral pre-rating (*M* = 1.37 € ± 0.06 SEM) vs. neutral post rating (*M* = 1.32€ ± 0.07; *t*(31) = 1.62, p = 0.11)). The change in congruent ratings (*M* = 0.025 ± 0.02 SEM) was however significantly different to the change in the neutral ratings (*M* = −0.05 ± 0.03 SEM; *t*(31) = 2.98, *p*_*corr*_ = 0.018) (Fig. 3C). The t-test between the change in congruent and incongruent ratings (*M* = −0.03 ± 0.03 SEM; *t*(31) = 2.04, *p*_*corr*_ = 0.15) and incongruent and neutral ratings (*t*(31) = 1.06, *p*_*corr*_ = 0.9) did not survive Bonferroni correction. Although the gaze cueing conditions differed in their initial ratings in both the taste and WTP dimensions, it is important to note that there was a significant interaction effect between the gaze cue and time in the WTP dimension indicating that the gaze cue had an influence on participants post ratings.

**Figure 4:**
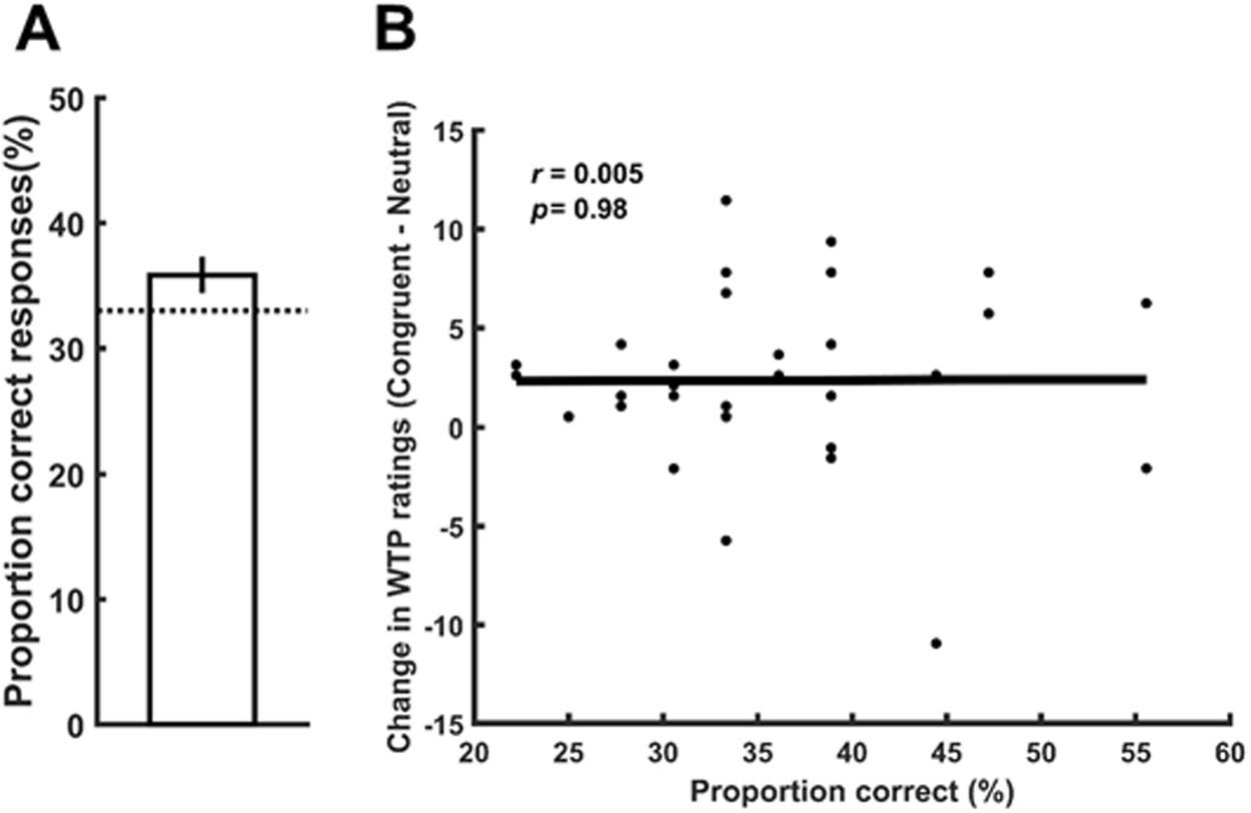
Contingency awareness. (**A**) No significant difference in task performance from chance level of 33%. Error bars denote within-subject SEM. (**B**) The increase in WTP in the congruent condition was independent of participants’ awareness of the contingency between the eye gaze and snack product (Pearson’s correlation). WTP, willingness to pay.

In order to probe whether the effect of congruence on WTP ratings was dependent on participants’ awareness of the contingency between the gaze and snack item, participants’ performance in the discrimination task was tested. A one-sample t-test (against the chance level of 33%) of participants’ performance did not reveal a significant difference from chance (*M* = 35.85% ± 1.45 SEM; *t*(31) = 1.76, *p* = 0.09) (Fig. 4A). In addition, there was no correlation between the difference in the change in WTP ratings for the congruent vs. neutral condition and participants’ discrimination performance (*r* = 0.005, *p* = 0.98) indicating that the increase in WTP occurred unconsciously, i.e., without participants’ awareness of the contingency between the gaze cue and product presentation (Fig. 4B).

### Analysis of eye-tracking data

**Figure 5:**
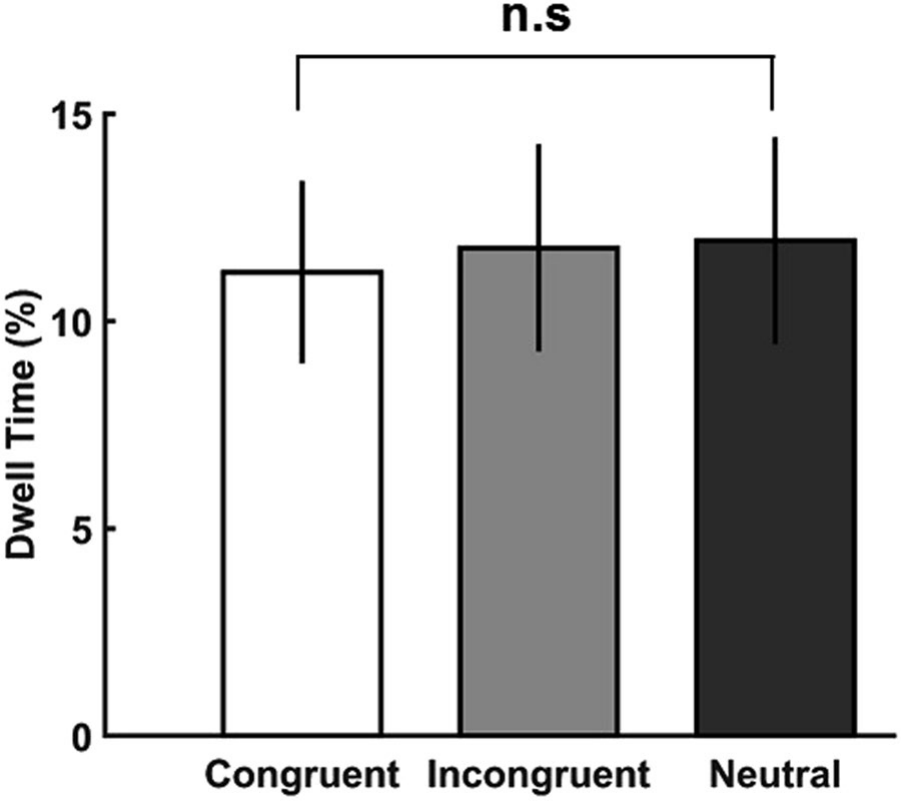
Dwell Time. No significant differences in the time spent looking at the food item in the three congruence conditions. Error bars denote within-subject SEM; n.s., not significant.

Finally, we analysed participants’ eye movements during the time interval in which the product was presented in the gaze phase (phase 2). To this end, we performed a repeated-measures ANOVA with the within-subject factor congruence for the dwell time on the food stimulus in each gaze condition. On average, 71.4% ± 4.8 SEM trials were included in the analysis. Analysis of the eye movements revealed that the mean dwell times in all three conditions were similar (congruent: *M* = 11.2% ± 2.2 SEM; incongruent: *M* = 11.8% ± 2.6 SEM; neutral: *M* = 11.9% ± 2.5 SEM) (Fig. 5). Thus, there were no significant differences between the three conditions (*F*(2,54) < 1, *p* > 0.5) suggesting that changes of individual preferences for food items depended on the social cue of someone else’s gaze, but not on participants’ increased overt attention to those food items.

Interestingly, while only around 11% of participants’ eye movements were spent on the food stimulus, approximately 77% of fixations landed on the central cue face (congruent: *M* = 77.9% ± 4.4 SEM; incongruent: *M* = 77.2% ± 4.8 SEM; neutral: *M* = 76.1% ± 4.4 SEM). Although, there were no differences in the dwell time on the cue face between the conditions (*F*(2,54) = 1.82, *p* = 0.17), there was a significant difference between the dwell time on the cue face vs. the food stimulus (*p* < 0.001), suggesting that the cue face may have been more salient than the food stimulus^33,34^.

#### Saccades

In a second step, we further analysed the number of saccades the participant made to the food item while it was presented. An rmANOVA with the factor gaze for the proportion of total saccades (congruent: *M* = 48.57% ± 4.47 SEM; incongruent: *M* = 53.96% ± 4.45 SEM; neutral: *M* = 50.07% ± 23.79 SEM) that were directed to the food item in the interval in which it was presented, revealed no main effect of the gaze cue (*F*(2,54) = 1.09, *p* = 0.34). Furthermore, there was also no significant main effect of gaze for the proportion of initial saccades (congruent: *M* = 34.96% ± 3.34 SEM; incongruent: *M* = 41.71% ± 3.68 SEM; neutral: *M* = 38.86% ± 3.39 SEM) that landed on the food item (*F*(2,54) = 3.05, *p* = 0.06). In general, participants seemed to make saccades on only one-third of all trials in each condition (96 trials per gaze condition; congruent trials *M* = 28.65% ± 4.25 SEM, incongruent trials *M* = 29.65% ± 4.21 SEM, neutral trials *M* = 33.48% ± 4.26 SEM). Together with the analysis of dwell times, this suggests that a majority of trials were spent fixating on the central cue face.

## Discussion

In the current study, we investigated whether gaze-evoked shifts in participants’ attention changed their explicit judgements and economic preferences of previously unknown food items. Although no changes in participants’ taste and health judgements for food items that were consistently looked at in comparison to those that were ignored by another person were observed, participants’ WTP for food items that were the focus of another’s attention was significantly higher than their WTP for unattended food items. Furthermore, participants’ eye-tracking data and their performance in a discrimination task suggest that the increase in WTP occurred independently of their overt attention to the food item and their knowledge of the contingency between the gaze cue and the target food item.

Our choices are largely dictated by the attention and therefore the value we assign a food item^35^. Indeed, our decision to select a food item is largely driven by its hedonic characteristics, which in turn drive attention^6^. The eyes are a powerful indicator of where attention is directed. Following another person’s gaze, an ability critical to normal social development^36^, has been shown to increase the likability of objects that are the focus of another’s attention^37^. In line with this, in the current study, participants were indeed willing to pay more money for items that were consistently looked at compared to items that were never looked at. Although there is a vast literature on the liking effects of objects induced by the attentional shifts from gaze cues^17–19^, our results show that such social-cue-evoked shifts in attention also alter participants evaluation of food items. Such factors that affect food choices are not only critical to understand abnormal food choices, which is a major factor in obesity^4^, but also play a role in consumer and marketing psychology^23^. Although the absolute change in participants’ WTP bids was itself not very high, the effect size reported in the current study is in line with previous studies investigating gaze cueing and object evaluations which reported effect sizes between 0.17 and 0.19^15,38^ and furthermore, the increase in bids was systematic across participants. It is important to note that the actual retail value of the food items itself was not very high. Thus, it would be interesting to investigate the effects of the gaze cue on more valuable items with greater variations in their retail price.

In addition to attentional shifts altering food preferences, implicit attitudes have also been shown to influence our choice for food items^39^. Specifically, Tang and colleagues (2014) observed that despite participants’ poor explicit judgements of the caloric content of food items, the reward value of a food item and their WTP preferences were dependent on implicit knowledge of caloric content^39^. In a similar vein, in the present study, the increase in WTP for congruent items occurred implicitly. In other words, although WTP increased for items that were looked at, participants were unaware of the association between the food item and the gaze cue (i.e. the contingency between the central face stimulus and a specific food item).

Previous research linked the role of attention in increasing value by demonstrating an increased dwell time or fixation for the later chosen product before the actual choice^7^. However, the reverse may also be true, i.e., an increase in value might drive a prolonged dwell time. However, in the current study, there was no difference between the dwell times for food items in the different conditions. On the one hand, our results suggest that the increase in WTP ratings for items that were gazed at were not driven by an increase in dwell time to the food item. On the other hand, it is interesting to note that participants only spent around one-tenth of the trial actually looking at the product (see Results: Analysis of eye tracking data). This could have been due to the following aspects of our task: (i) participants performed a relatively easy detection task, which did not require them to dwell on the product to perform the task, (ii) the central presentation of the face which may have acted as a distractor and (iii) the low eccentricity of the product from the central fixation. Thus, participants could easily respond to the appearance of the product without performing eye movements, resulting in low dwell times on the product. Indeed, it has previously been shown that total fixation duration is dependent on the task and task instruction^40^. Finally, the central face was always present even after the appearance of the food item. It is well known that faces are salient biological stimuli and our attention is preferentially directed to faces rather than to objects in a scene^41,42^. Accordingly, the central cue face may have acted as a salient distractor and diverted attention away from the food item. Indeed, it has previously been shown that faces compete for attention when presented with non-face stimuli^34^. In line with this, participants spent a significant proportion of time fixating on the central cue face. Based on the design of the current task it therefore remains unknown whether the observed eye tracking results would be stronger if the task is modified such that more attention is directed towards the product. The continuing presence of the central cue face along with the food item could also explain the intriguing pattern of results from the cueing task. While participants’ were faster to respond to food items that appeared at the gazed at location, compared to the incongruent gaze condition, participants were slowest in the neutral condition. A large body of previous research shows that direct gaze has profound effects on the receiver, not only capturing and guiding visual attention but also delaying the disengagement of attention^43,44^. Thus, attentional disengagement from the cueing face in the neutral condition could have resulted in slower reaction times to the food item. It is however, important to note that most studies on gaze cueing typically have only a congruent or incongruent condition^16,45^. The addition of the neutral condition in the current study was intended as a control. An interesting question raised by our results for future studies is, whether this change in WTP for congruent food items, measured behaviourally, may have caused a concurrent increase in the neural responses to the same items.

Interestingly, WTP has been shown to be related to health and taste attributes^46^. However, in the current study, we found no gaze-evoked changes in participants’ health and taste ratings for the food items which can be explained by certain factors in the design of the task. Firstly, the face stimuli that were used as gaze cues in the task had neutral expressions. The task may have been more sensitive to changes in the taste and health dimensions if the face cues displayed an emotion. Analogously, Bayliss and colleagues found that affective evaluations of objects are indeed influenced by the emotional expression of the cue stimulus^15,47^. Furthermore, providing nutrient and taste information has been shown to increases the willingness to try novel food^48^ as well as influences participants’ WTP^49^. As the food items in the present study were unfamiliar to the participants, it is plausible that participants’ beliefs and consequently their ratings were noisier on these dimensions and providing participants with taste and health information in combination with the gaze cue with an emotional expression could have changed their judgements on these dimensions.

Taken together, in the current study, we observed an implicit influence of the gaze cue on the WTP. Such an implicit and unconscious change in the likability of objects that were the focus of attention has also found previously^39^. However, in contrast to previous studies which required participants to explicitly identify the object, in the current study participants performed a low-level localization task. Thus it is plausible that covert attention plays a major role in this change in value through the gaze cue. The current results complement those of a novel eye tracking study investigating the influence of gaze cues on attention to print advertisements^23^. Specifically, the reader’s attention to the product and brand in the advertisement increased when a model was looking at the product being advertised compared to when the model looked at the reader^23^. Despite the contradictory results in the health and taste dimensions, the current findings of an implicit increase in WTP through a gaze-evoked shift in attention, provide an interesting platform for the fields of consumer psychology and neuromarketing.

## Methods

### Participants

Thirty-two participants (20 females; mean age: 23.9 (±0.67 SEM) years)) were invited to take part in the study. All participants had normal or corrected-to-normal vision, were naïve to the purpose of the study, and monetarily endowed for their participation. The study was conducted in accordance with the 2008 World Medical Association Declaration of Helsinki and was approved by the local ethics committee of the University of Lübeck. Written informed consent was obtained from all participants before the experiment.

### Stimuli

The food stimuli consisted of 36 Korean snack products that were unknown to all participants. All food stimuli were cropped to a size of 12° × 13° degrees of visual angle, were displayed on a grey background and matched to contain an equal number of informative pixels. For the cueing phase, 4 neutral faces (2 male) from the Radboud Face database^50^ were selected. The faces were displayed on a grey square that matched the monitor background and the food stimulus background and comprised a square of 17° × 17°. All visual stimuli were presented with Matlab 2017b (The MathWorks, Natick, MA, USA), using the Psychtoolbox 3 (http://psychtoolbox.org/) on a grey background and displayed on a 27-inch CRT monitor (resolution: 1920 × 1080 Px; refresh rate: 120 Hz).

### Design and procedure

**Figure 6:**
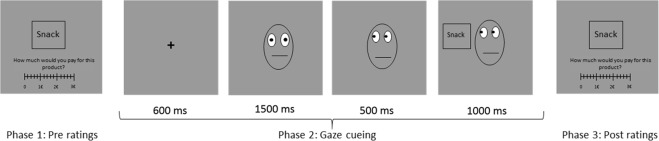
Task Structure with a depiction of a congruent trial in phase 2. The experiment was divided into three phases (pre-rating, gaze-cueing and post-rating phase). In the pre- and post-rating phase, participants rated, at their own pace, each food item for their health, taste and WTP. Participants’ change in preferences were calculated based on their ratings from phase 1 and 3. In the gaze-cueing phase, each food item was associated with a gaze direction to investigate the influence of the gaze-cue (social cue) on food preferences. The face stimuli depicted here is a schematic representation. Stimuli used in the experiment were obtained from the Radboud Face database^50^ (http://www.socsci.ru.nl:8180/RaFD2/RaFD).

The experiment involved three phases: a pre-rating, gaze-cueing and post-rating phase (Fig. 6). Throughout all phases, participants viewed the computer screen located 50 cm away and their head was stabilized by a chin rest. Eye movements were recorded during all three phases with a high-speed video-based eye-tracker (Eyelink 1000, SR-Research, Ontario, Canada; sampling rate: 500 Hz).

Participants were instructed at the beginning of the experiment that they would receive 8 €/hour for their participation as well as 3 € additionally to buy one of the snack items. The snack items were offered using a Becker–DeGroot–Marschak (BDM) auction^47^, where participants place a bid on each item. Participants were instructed before the auction that one trial would be chosen at random at the end of the experiment. If the bid made by the participant exceeded a random number drawn with equal probability from a known distribution, the participant paid the amount drawn from the distribution. Else, the participant did not get the product and did not pay anything. This ensured that every trial was treated as unique and participants did not have to worry about using the 3 € budget over different items. Such a BDM auction has been shown to be a good indicator of participants’ willingness to pay (WTP), and thus individual preference, for an item^29,30,51^.

Thus, in the first (pre-rating) phase of the experiment, participants rated the 36 Korean snack items on three dimensions: taste and health on a 7-point Likert-scale (1 = *not at all tasty/healthy*, 7 = *very tasty/healthy*) and their WTP for the product. WTP bids were made as explained above on a scale ranging from 0 to 3 € in 20 cent increments. After participants’ initial ratings, the products were sorted into high, medium and low categories (12 products in each category) based on their WTP bids.

The pre-rating phase was followed by the gaze cueing phase (Fig. 6). Each trial in the cueing phase began with a 600 ms fixation cross which was then replaced by a neutral face gazing straight ahead. The face stimulus was presented for 2 s after which one of the food stimuli was presented, either to the left or the right of the face stimulus. In two-thirds of the trials, 1.5 s after the onset of the central cue stimulus, the central cue face made a gaze shift, either to the left or the right. In congruent trials, the gaze of the face was directed towards the food product while in incongruent trials the gaze of the face stimulus was directed away from the food stimulus. In the remaining one-third trials (i.e. the neutral trials), no gaze shift occurred, and the central cue face continued gazing straight ahead. Participants’ task was to indicate as quickly as possible via button press where the food stimulus appeared, i.e., to the left or the right. They had 1.5 s to respond. An equal number of high, medium and low rated products were divided into the three cueing categories: congruent, incongruent and neutral. It is important to note that each food product was associated with a fixed gaze contingency, i.e., congruent, incongruent or neutral throughout the whole experiment. However, each food item was paired with the different faces. This association between the product and the gaze condition was counterbalanced across participants. Participants performed 8 runs of 36 trials each, such that there were 8 repetitions of each food product combined with gaze before the final post-rating phase.

In the post-rating phase, participants again rated the snack products on the same three dimensions as in the pre-rating phase: taste, health and WTP. In addition, there was a fourth question, which probed participants’ awareness of the contingency between the gaze cue and the snack item. Specifically, participants were asked in a discrimination task to indicate the direction of eye gaze when a snack item was presented, with the options to answer as ‘at the product’ (congruent condition), ‘away from the product’ (incongruent condition), or ‘straight ahead’ (neutral condition). All ratings in the pre-rating and post-rating phase were self-paced. Finally, participants played the auction to see whether and which snack they will be endowed with.

### Data analyses

#### Analyses of behavioural data

For the analysis of the congruence effect during the gaze task (phase 2), mean reaction times in all correct trials for each condition were computed and subjected to a one-way repeated measure analysis of variance (ANOVA) with congruence as within-subject factor. The change in participants’ ratings after the gaze task was computed for each food item and each condition using eq. (1):

1 $$\frac{100\ast (Post\,rating\mbox{--}Pre\,rating)}{length\,of\,scale}$$

Thus, a positive value indicates that participants’ rating for the product increased after the gaze task (phase 2). In a second step, these values were mean-centred and analysed by means of one-way repeated measures ANOVA. Post-hoc t-tests to explore interaction effects were corrected for multiple comparisons using Bonferroni correction. *p*_*corr*_ refers to the Bonferroni-corrected p value, corrected for the number of comparisons.

Participants’ awareness of the contingency between the gaze cue and the product was assessed based on the proportion of correct responses in the discrimination task. When performance in this task does not differ from the chance level of 33%, participants are considered to be unaware of the relation between the product and the gaze shift. A one-sample t-test against the chance level of 33% was computed to test this contingency awareness. In addition, Pearson’s correlation was performed between participants’ performance in the discrimination task and their change in ratings in the congruent condition to test whether the gaze-cueing manipulation was dependent on the awareness of the relationship between the gaze cue and the snack item.

### Analyses of eye-tracking data

Eye movements were analysed during the gaze task (phase 2) for all trials in which at least 95% of data were available and not lost due to artefacts (example, blinks) (cf.^13^).

For these included trials, the dwell time on the stimulus during the 1.5 s interval, in which the snack stimulus was presented, was computed for each gaze condition to determine whether more attention was directed to the stimulus in the congruent vs. incongruent and neutral conditions. Four participants were excluded either due to problems during data acquisition (1) or poor eye-tracking data quality (3). Thus, this analysis was based on 28 participants. In addition, we computed the proportion of initial saccades and proportion of total saccades that were directed to the food product in each gaze condition. To this end, eye movements were identified as saccades if the velocity of at least 8 (16 ms) consecutive data points exceeded 60°/s.

## Data Availability

The datasets generated and analysed during the current study are available from the corresponding author on reasonable request and are on the open science framework (osf: https://osf.io/kgsbf/).
